# First-in-men experience with a novel frozen elephant trunk prosthesis featuring an endovascular side branch for left subclavian artery connection

**DOI:** 10.1093/ejcts/ezae302

**Published:** 2024-08-12

**Authors:** Sandra Folkmann, Zsuzsanna Arnold, Daniela Geisler, Verena Lenz, David Miosga, Marieluise Harrer, Hubert Trnka, Rene Eller, Thomas Aschacher, Bernhard Winkler, Martin Czerny, Gabriel Weiss, Martin Grabenwöger

**Affiliations:** Department of Cardiovascular Surgery, Clinic Floridsdorf, Vienna, Austria; Karl Landsteiner Institute of Cardiovascular Research, Vienna, Austria; Department of Cardiovascular Surgery, Clinic Floridsdorf, Vienna, Austria; Karl Landsteiner Institute of Cardiovascular Research, Vienna, Austria; Department of Cardiovascular Surgery, Clinic Floridsdorf, Vienna, Austria; Karl Landsteiner Institute of Cardiovascular Research, Vienna, Austria; Department of Cardiovascular Surgery, Clinic Floridsdorf, Vienna, Austria; Karl Landsteiner Institute of Cardiovascular Research, Vienna, Austria; Department of Cardiovascular Surgery, Clinic Floridsdorf, Vienna, Austria; Karl Landsteiner Institute of Cardiovascular Research, Vienna, Austria; Department of Cardiovascular Surgery, Clinic Floridsdorf, Vienna, Austria; Karl Landsteiner Institute of Cardiovascular Research, Vienna, Austria; Department of Radiology and Interventional Radiology, Clinic Floridsdorf, Vienna, Austria; Department of Radiology and Interventional Radiology, Clinic Floridsdorf, Vienna, Austria; Department of Cardiovascular Surgery, Clinic Floridsdorf, Vienna, Austria; Karl Landsteiner Institute of Cardiovascular Research, Vienna, Austria; Department of Cardiovascular Surgery, Clinic Floridsdorf, Vienna, Austria; Karl Landsteiner Institute of Cardiovascular Research, Vienna, Austria; Sigmund Freud Private University, Medical Faculty, Vienna, Austria; Department of Cardiac and Vascular Surgery, University of Freiburg, Freiburg, Germany; Department of Cardiovascular Surgery, Clinic Floridsdorf, Vienna, Austria; Karl Landsteiner Institute of Cardiovascular Research, Vienna, Austria; Sigmund Freud Private University, Medical Faculty, Vienna, Austria; Department of Cardiovascular Surgery, Clinic Floridsdorf, Vienna, Austria; Karl Landsteiner Institute of Cardiovascular Research, Vienna, Austria; Sigmund Freud Private University, Medical Faculty, Vienna, Austria

**Keywords:** Aortic disease, Frozen elephant trunk, Left subclavian artery side branch, Cerebral perfusion technique, Distal aortic perfusion

## Abstract

**OBJECTIVES:**

The objective of this study was to enhance the efficiency of aortic arch replacement through the development of a novel frozen elephant trunk (FET) prosthesis with an endovascular side branch for left subclavian artery (LSA) connection. After successful pre-clinical testing, the feasibility and safety of implementing this innovative prosthesis in human subjects were investigated.

**METHODS:**

Between September 2020 and September 2021, 4 patients (mean age 67) with conditions such as penetrating ulcer, non A–non B aortic dissection and chronic arch aneurysm underwent surgery utilizing the customized device. Surgeries were performed under high moderate hypothermia (27°C), employing bilateral selective antegrade cerebral perfusion (SACP) and distal aortic perfusion. Anastomosis of the FET prosthesis with the aortic arch occurred in zone 1, followed by separate reimplantation of the left common carotid artery and the brachiocephalic artery.

**RESULTS:**

All patients were discharged in good clinical condition. The mean aortic cross-clamp, antegrade selective cerebral perfusion and distal aortic perfusion times were 111, 71 and 31 min, respectively. Endovascular extension of the side branch for the LSA was required in all cases to prevent endoleak formation. One patient received a stent graft extension at the end of the operation, while 2 others underwent the procedure during their hospital stay. One patient was diagnosed with an endoleak at the first follow-up after 3 months, and endoleak sealing was achieved via the brachial artery with an extension stent graft.

**CONCLUSIONS:**

Preliminary clinical outcomes suggest that the newly designed FET prosthesis shows promise in simplifying total arch replacement. These initial findings provide a foundation for planned clinical studies to further assess the effectiveness of this modified surgical hybrid graft, with particular attention to the length and diameter of the LSA sidearm.

## Graphical abstract

**Figure:**
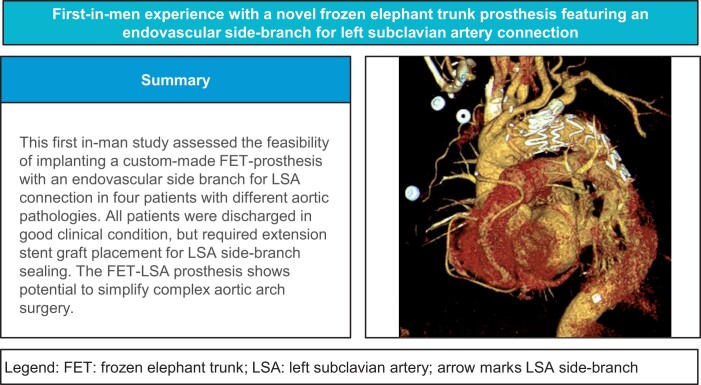

## INTRODUCTION

Over the past 15 years, the frozen elephant trunk (FET) technique has become an important treatment approach for managing complex aortic arch aneurysms as well as acute and chronic aortic dissections [1–5]. Ongoing enhancements to both the hybrid prosthesis and the surgical technique have resulted in better outcomes and expanded utilization of this method [6, 7]. Key elements contributing to this favourable progress include the implementation of high moderate hypothermia coupled with selective antegrade cerebral perfusion (SACP), proximalization of the distal anastomosis in zone 2 and restrictions on the length of the stent graft [8]. The ongoing enhancement of FET devices, along with advancements in surgical techniques, has yielded promising outcomes in managing complex aortic arch pathologies. Consequently, the utilization of the FET technique for specific indications has been incorporated into the latest guidelines for addressing acute and chronic syndromes of the aortic organ [9].

Nevertheless, a surgical challenge persists, namely, the anastomosis to the left subclavian artery (LSA). Various methods have been recommended to tackle this issue [10–12]. In a pre-clinical experimental study, we were already able to show the viability of utilizing an endovascular side branch to connect with the LSA [13]. This study outlines the outcomes of the initial 4 patients who underwent treatment with a custom-made FET prosthesis that incorporates an endovascular side branch for connecting to the LSA.

## PATIENTS AND METHODS

### Patients

**Figure 1:**
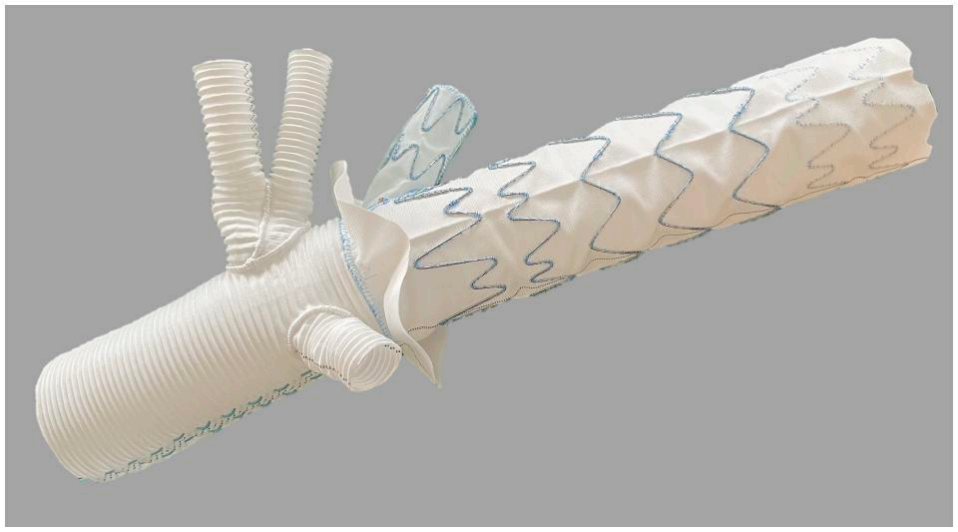
Custom-made endovascular stent graft with a stented side branch for LSA connection.

The study was approved by the local Ethics Committee Nr. EK/24-038-VK of the City of Vienna/Austria and registered as an observational study. All patients gave their informed consent prior to inclusion in the study. Between August 2020 and September 2021, patients with aneurysms or dissections of the aortic arch and proximal descending aorta underwent aortic arch replacement with a custom-made FET prosthesis with an endovascular side branch for LSA connection. The aim of this prospective observational study was to assess the feasibility of implantation and the safety of this novel device (Fig. 1). Patient selection for this first-in-men study was based on both anatomical and patient-specific criteria. Morphological contraindications for the use of this device include an aneurysmatic or stenotic LSA or severe kinking of the LSA. Additionally, a separate take-off of the left vertebral artery from the aortic arch also prevents the use of this device. Only stable patients with normal cardiac and renal function, who could wait the 4–6 weeks required for the manufacturing process of this custom-made device, were selected for this initial study.

The following outcome parameters were analysed: hospital mortality, new disabling stroke, new paraplegia, new paraparesis and permanent dialysis (>90 days). These parameters were evaluated at 2 different time points: after the intervention to discharge visit and at the 6-month visit.

### Custom-made FET prostheses with LSA side branch

**Figure 2:**
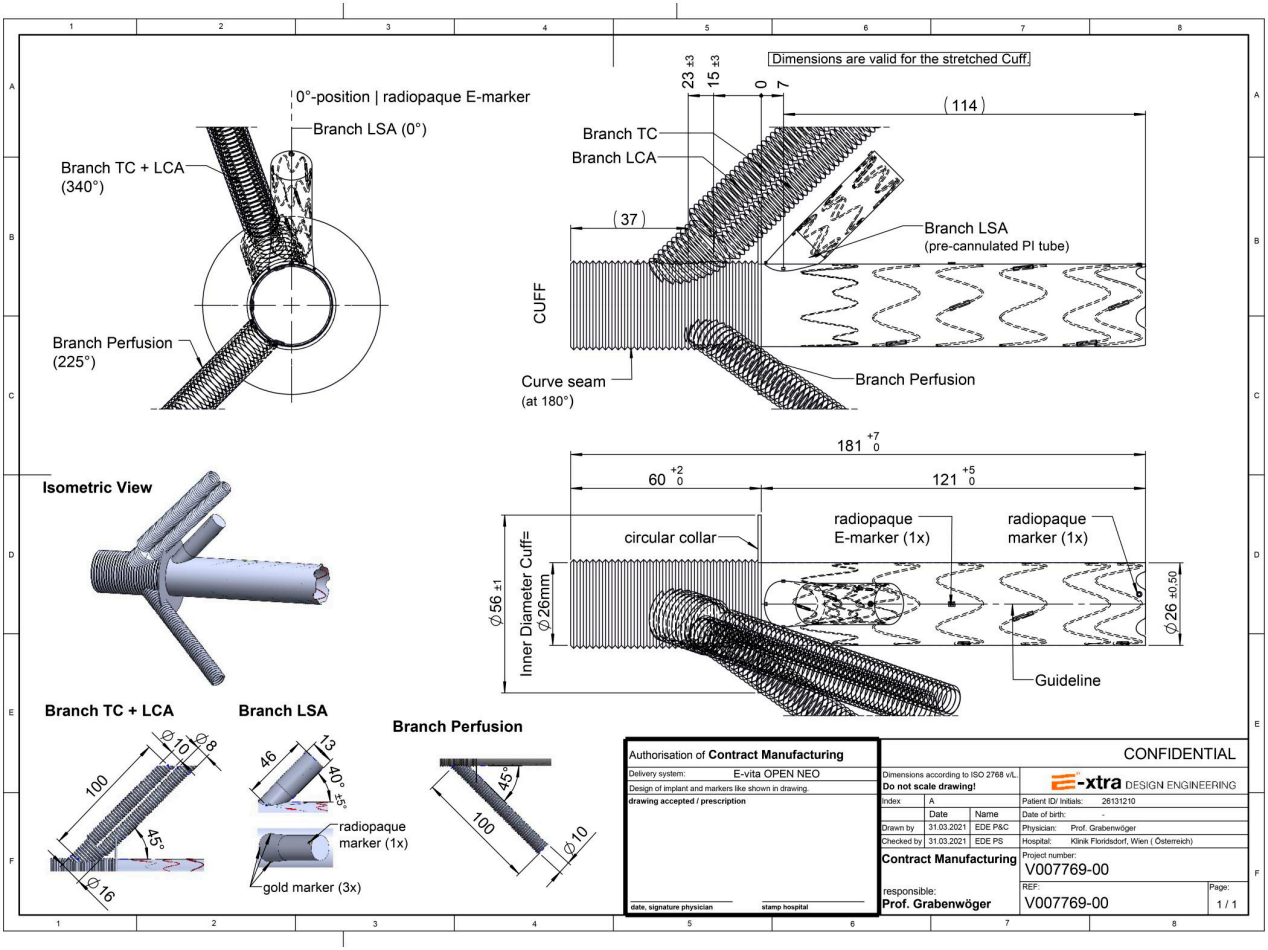
Technical drawing of a custom-made FET–LSA prosthesis prior to the manufacturing process.

The FET–LSA prostheses were individually tailored for each patient based on preoperative computed tomography (CT) scans according to a technical drawing (Fig. 2). These devices were manufactured by Jotec (Jotec/Artivion, Hechingen, Germany) and purchased by the hospital (Fig. 1). Unfortunately, the clinical program had to be halted due to the implementation of European Medical Device Regulations (MDR), making it impractical to manufacture these custom-made devices. The FET–LSA prostheses were contract-manufactured devices, for which the treating physician was fully responsible. Due to the changed regulatory requirements of the MDR, the company has decided to place the products on the market as custom-made devices under the name of JOTEC (Jotec/Artivion, Hechingen, Germany) as a legal manufacturer. This process has not been yet finalized.

For patients with chronic aneurysmal disease, an oversizing of 10–15% was calculated, while no oversizing was applied for those with dissection. The standard length of the stent graft was 120 mm, but for 1 patient with mega-aortic syndrome, a stent graft length of 160 mm was chosen. The dimensions of the LSA side branch were 15 mm in diameter and the length varied between 35 and 46 mm. The exact dimensions of the custom-made FET–LSA prostheses are listed in Table 1.

**Table 1:** Size specifications of the custom-made FET–LSA prostheses and the Advanta extension stent grafts für LSA endoleak sealing

	Ø Dacron prosthesis (mm)	Ø main stent-graft (mm)	Length stent-graft (mm)	Ø LSA branch (mm)	Length of LSA-branch (mm)	Advanta stent-grafts (mm)	Viabahn VBX stent-graft (mm)	LSA diameter (mm)
# 1	30	30	120	13	35	9 × 38		9
# 2	30	30	160	13	46		8 × 39	11
# 3	26	24	120	13	35	9 × 38		8
# 4	28	28	120	13	35	9 × 38		9

### Surgical technique

In all patients, surgical access was obtained via a full median sternotomy. At the start of the procedure, a guide wire was passed through the left brachial artery into the aortic arch to aid in the subsequent wire-guided insertion of the endovascular LSA side branch into the LSA itself. Extracorporeal circulation was initiated by cannulating the right axillary artery and the right atrium. Selective antegrade bilateral cerebral perfusion (SACP) was consistently employed in every case. Upon establishment of cardiopulmonary bypass (CPB), the patient’s core body temperature (measured via bladder and rectal probes) was gradually reduced to achieve high moderate hypothermia (27°C). Once the desired temperature was attained, CPB was temporarily halted and the aortic arch was opened. Bilateral SACP was maintained with a total flow rate of 10 ml/kg body weight, achieved by clamping the brachiocephalic trunk directly distal to its branching from the aortic arch and by inserting a perfusion line into the left common carotid artery (LCCA). Cerebral oxygen saturation was continuously monitored throughout the entire procedure using near-infrared spectroscopy (NIRS). The take-off of the LCCA was ligated. The ascending aorta, as well as the proximal aortic arch, was resected in zone 1, with special attention to the concavity of the aortic arch, which needed to be preserved. Because of the presence of the LSA side branch, the collar of the FET prosthesis for distal anastomosis is positioned very proximally, necessitating the use of aortic tissue in the lesser curvature of the arch to complete the anastomosis. The location of the distal arch anastomosis is determined by the distance between the LSA side branch and the collar or the FET prosthesis.

The stent graft implantation was conducted with the support of a guide wire, which was advanced antegradely through the opened aortic arch into the descending aorta, alongside the previously positioned guide wire in the LSA. Positioning of the guide wire in the descending aorta was controlled by angioscopy. Initially, the LSA branch was deployed, followed by the main body of the FET prosthesis. Upon completing the distal anastomosis in zone 1, de-airing of the prosthesis was performed and distal aortic perfusion via the side branch was initiated with 3.5–4.5 l/min. The pressure in both the femoral artery and the left radial artery was maintained within the range of 40–50 mmHg. Cerebral and distal aortic perfusion were conducted using a single pump head and a Y-connector in the arterial line. Following the completion of the end-to-end anastomosis between the LCCA and the innominate artery, the process of rewarming commenced and subsequently, the anastomosis with the proximal aorta was executed. The whole surgical procedure is demonstrated in a live-in-a-box video (Supplementary Material, Video S1).

### LSA stent graft extension

To address the LSA side branch endoleaks, we utilized extension Advanta^®^stent-grafts (Getinge, Rastatt, Germany) as well as Viabahn^®^ VBX^®^ stent grafts (W.L. Gore & Associates, Inc., Newark, DE, USA) delivered through the left brachial artery for sealing. The diameter and length of these devices are listed in Table 1.

### Follow-up protocol

A clinical follow-up examination and assessment of the aorta via CT-scans were conducted before discharge, at 6 and 12 months following the operation and then annually thereafter.

### Statistical analysis

Categorical variables were presented as frequency (percentage), while continuous variables were expressed as mean ± standard deviation (range). Statistical analysis was not conducted due to the limited number of patients in this case series.

## RESULTS

Between August 2020 and September 2021, 4 patients (2 female) with a mean age of 67 years (range 50–79 years) underwent aortic arch replacement with a custom-made FET-prosthesis with an endovascular side branch for LSA connection.

**Figure 3:**
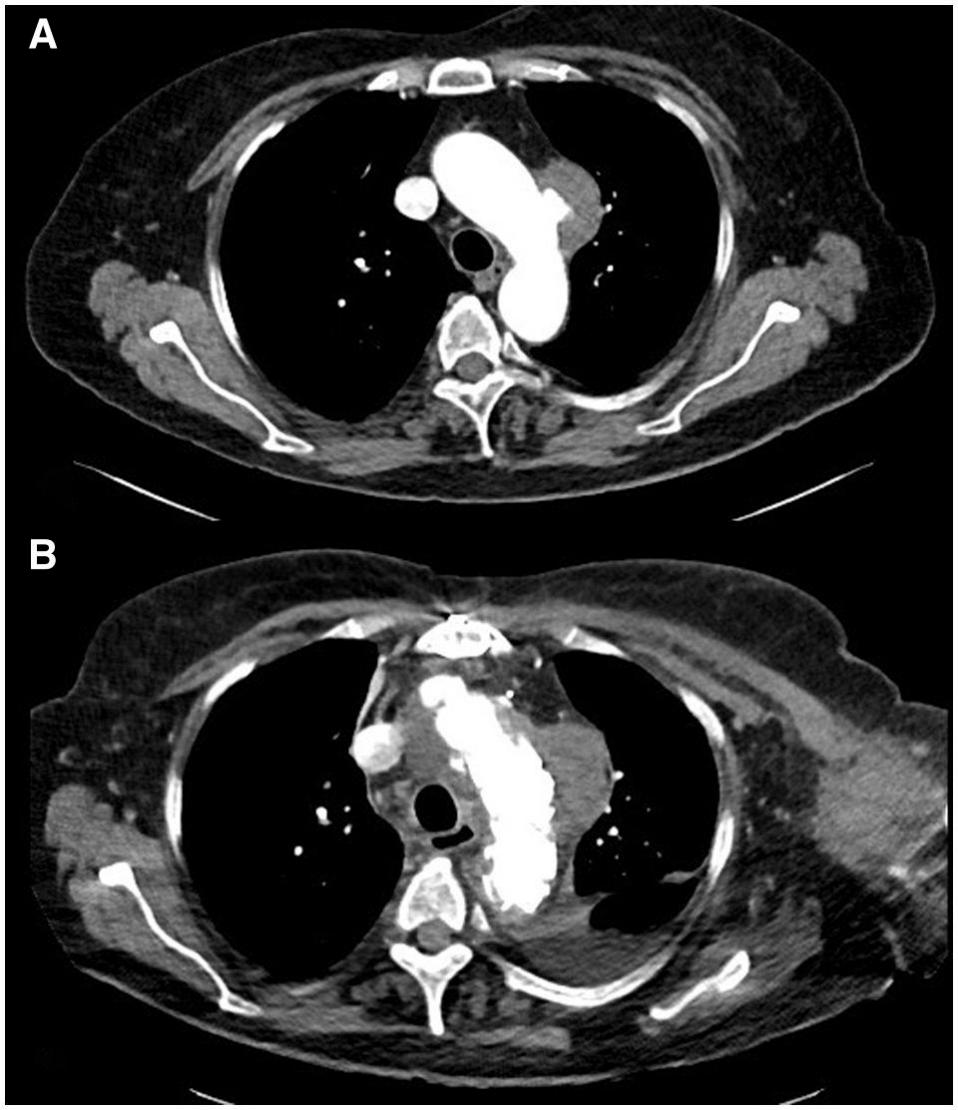
(**A**) Penetrating aortic ulcer located in the distal aortic arch with a diameter of 4.5 cm and (**B**) complete exclusion of the PAU after FET–LSA operation.

**Figure 4:**
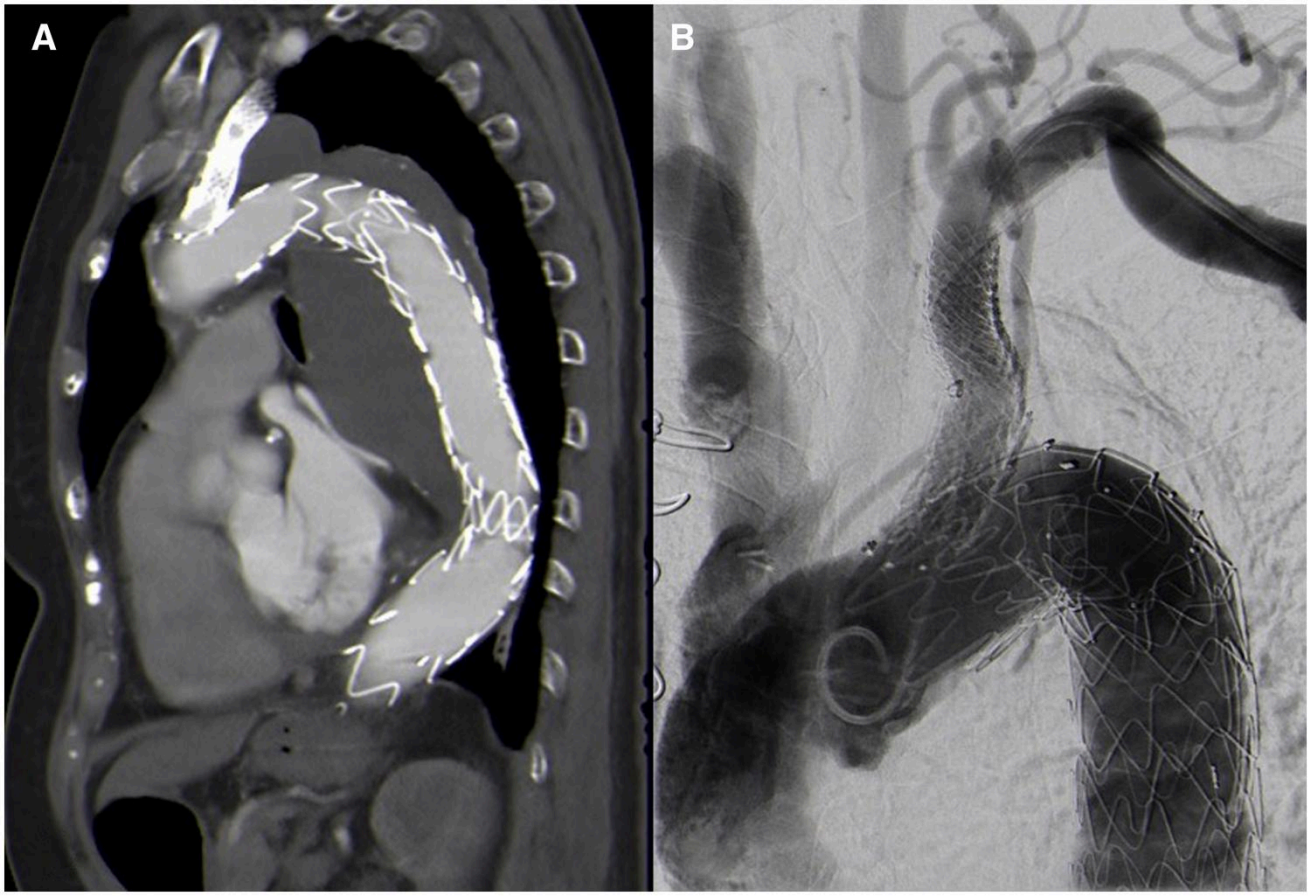
(**A**) Aneurysm of the distal aorta and descending aorta excluded by the FET–LSA prosthesis after placement of extension stent graft in the LSA and distal descending aorta and (**B**) angiography demonstrated complete exclusion of the aneurysm after placement of Advanta extension stent graft in the LSA.

The patient’s details are presented in Table 2. In 1 patient, the indication for surgery was a penetrating atherosclerotic aortic ulcer (PAU) with a diameter of 4.5 cm located in the distal aortic arch (Fig. 3A and B), while in another patient, it was a subacute non-A–non-B aortic dissection. Additionally, 2 patients presented with a chronic atherosclerotic aneurysm affecting the distal aortic arch and proximal descending aorta (diameter 5.4 and 6.5 cm). Two patients necessitated a simultaneous bypass graft procedure to the left anterior descending coronary artery (LAD). In the case of the patient diagnosed with mega-aortic syndrome, a plan was made to undergo subsequent endovascular stent graft extension during the postoperative period (Fig. 4A and B).

**Table 2:** Patient’s demographics

Demographic, risk factors and comorbidities		Study population *n* = 4
Age (years) (range)	67	(50–79)
Female, *n* (%)	2	(50)
Body mass index (BMI), *n* (range)	25.75	(21.1–30.1)
Adipositas (BMI >30), *n* (%)	1	(25)
Smoker, *n* (%)	3	(75)
Hypertension, *n* (%)	3	(75)
Dyslipidaemia, *n* (%)	2	(50)
Chronic renal failure, *n* (%)	0	(0)
Diabetes, *n* (%)	2	(50)
COPD, *n* (%)	1	(25)
Positive family history, *n* (%)	1	(25)
CAD, *n* (%)	2	(50)
CVD, *n* (%)	1	(25)
PAD, *n* (%)	0	(0)
Add. EuroSCORE (range)	7	(3–8)
Log. EuroSCORE (range)	9.00	(2.8–10.3)
EuroSCORE II (range)	3.3	(1.8–4.3)

CAD: coronary artery disease; COPD: chronic obstructive pulmonary disease; CVD: cerebrovascular disease; PAD: peripheral arterial disease.

All patients were able to be discharged in good clinical condition. There were no instances of postoperative bleeding or neurological complications observed. Operative specifics, including CPB duration, antegrade cerebral perfusion time and distal ischaemia time, are presented in Table 3. The duration of postoperative ventilation did not surpass 24 h in any case. The mean intensive care unit stay was 4.8 days (ranging from 2 to 6 days), while the mean hospital stay duration was 25.6 days, with a range of 16–36 days. The prolonged hospital stay of 25.6 days can be attributed to 1 patient who developed a significant haematoma in the left arm following LSA stent graft extension and to another patient, who required subsequent thoracic endovascular treatment for the mega-aortic syndrome.

**Table 3:** Intraoperative perfusion details

Patients	CPB-duration	X-clamp time	SACP	Distal aortic perfusion	Visceral ischemia time
# 1	198	122	60	27	33
# 2	156	97	61	25	36
# 3	218	105	74	33	41
# 4	211	120	90	41	49
Mean value	195.75 ± 24.05	111 ± 10.42	71.25 ± 12.15	31.5 ± 6.22	39.75 ± 6.01

Endovascular extension of the LSA-side branch was necessary in all patients to cover the endoleak. In 1 patient, who was operated on in a hybrid operation room, extension stent graft placement was performed at the end of the operation. In 2 other patients, this intervention was carried out during their hospitalization. In 1 case, endoleak detection became apparent during the 3-month follow-up, subsequently repaired through endoleak sealing with an extension stent graft. A single patient required surgical intervention to address a pseudoaneurysm of the left brachial artery, resulting in a vascular complication following endovascular therapy. This complication resulted in a prolonged hospital stay. The patient diagnosed with mega-aortic syndrome underwent placement of 2 additional stent grafts (Relay^®^ pro; Terumo Europe, Leuven, Belgium) for distal extension, aiming to fully exclude the aneurysm located in the descending aorta (Fig. 4A). Two endovascular grafts were used with a diameter of 30 mm and a length of 164 mm.

The follow-up period for the patients varied between 27 and 40 months with a mean value of 35 months. All patients are examined in our outpatient clinic and are in good clinical health without any restrictions in their daily routine. Substantial shrinkage of the excluded PAU and distal arch aneurysm could be observed in 2 patients. Occlusion of the false lumen could be achieved in the patient with the aortic dissection till the end of the stent graft. In the patient with the mega-aortic syndrome, aortic diameter remains stable and a small type II endoleak originating from an intercostal artery cannot be excluded.

## DISCUSSION

The inaugural phase of this initial study in men may indicate the viability of employing a FET prosthesis with an endovascular side branch to link with the LSA. While extension stent grafts were required for all patients to ensure full sealing of the side branch to the LSA, this study does establish a proof of concept. All patients could be discharged in good clinical condition and are subjected to a strict follow-up protocol. The implementation of the European MDR prevented a continuation of the production of these custom-made devices and therefore this clinical program could not be continued. Nevertheless, valuable information on the applicability of this technique as well as the length and diameter of the side arm could be obtained.

The FET technique for total arch replacement has faced scrutiny due to prolonged operative and circulatory arrest times and higher neurologic complication rates [5, 14–16]. Despite this, refinements in the technique have led to its wider acceptance, particularly by proximalizing the distal anastomosis of the FET prosthesis in zone 2 of the aortic arch [8]. The anastomosis to the LSA remains a major challenge, especially in patients with a deep chest, as it can be time-consuming and extend the duration of antegrade cerebral perfusion. Various methodologies are advocated and endorsed by specialized institutions [10–12]. Methods to address this include a 2-stage approach with an initial subclavian to carotid artery bypass, unsuitable for urgent cases and an extra-anatomic transthoracic bypass from the LSA to the ascending aorta [17]. The direct anastomosis of the LSA to the side branch of the FET prosthesis can be challenging and time-consuming, particularly in obese patients with a posterior and deep anatomical position of the LSA.

**Figure 5:**
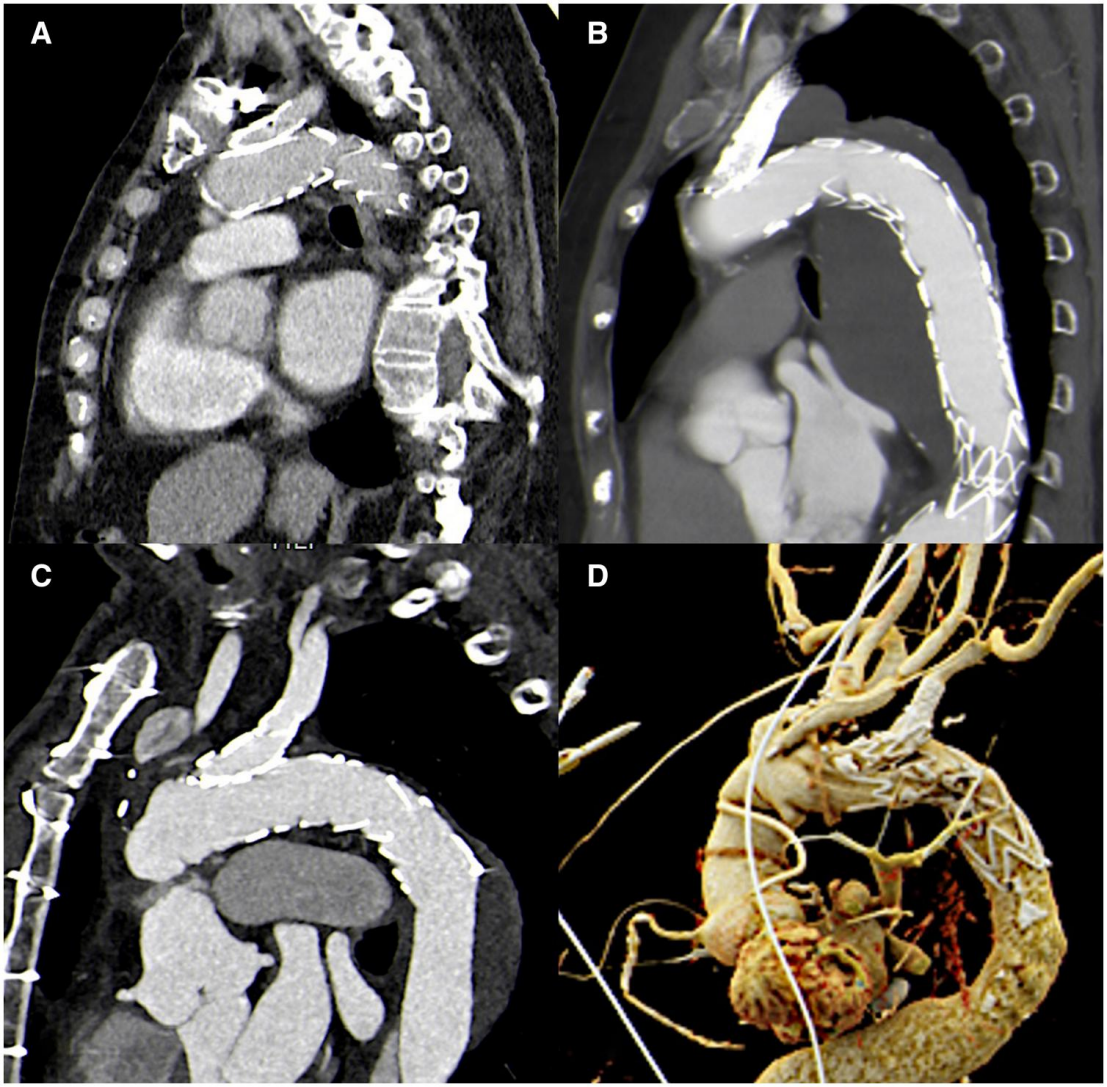
(**A–D**) Completion CT angiography scans focused on the LSA side branch of all patients; imperfect insertion of the side branch into the LSA can be observed in all cases as well as sealing of the LSA by extension stent grafts.

Therefore, establishing an endovascular link between the FET prosthesis and the LSA presents an appealing strategy for surmounting the challenges associated with surgical anastomosis. Two critical factors necessitate consideration to materialize this concept: the length and diameter of the LSA side branch. We have previously investigated these anatomical parameters in a pre-clinical experimental investigation, revealing that the mean separation between the emergence points of the LSA and the vertebral artery is 40.5 ± 9.3 mm, with a mean LSA diameter of 11.1 ± 3.8 mm [13]. Because all FET–LSA prostheses implanted in our initial series in humans were custom-made, the length and diameter of the LSA side branch were specifically adjusted to each patient (Table 1). The necessity for all patients to undergo an endovascular extension to address the LSA endoleaks can be attributed to certain technical challenges encountered in this prototype series. These challenges included instances where the retrieval of the introduction sheath resulted in the unintended retraction of the LSA side branch in 1 patient and early deployment of the LSA side branch in another patient. These challenges elucidate why the SACP time could not be significantly decreased compared to the procedure with conventional FET prostheses [1]. Additionally, there remains the question of whether the main body of the FET prosthesis should be deployed prior to the LSA side branch. In our series, we opted to deploy the LSA side branch first, a strategy that may be suboptimal as a portion of the side branch consistently remained within the aorta. Looking back, deploying the main body first would lead to a more optimal insertion of the side branch into the LSA. The imperfect positioning of the LSA-side branch may be attributed to technical difficulties with the prototype devices on 1 hand and the sequence of the deployment process on the other. Nonetheless, we were able to effectively address LSA endoleaks in all patients by utilizing extension stent grafts inserted through the brachial artery (Fig. 5). For this supplementary procedure, we utilized Advanta stent-grafts ranging in diameter from 9 to 10 mm and measuring 38 mm in length along with Viabahn VBX stent grafts with a diameter of 8 mm and a length of 39 mm. These stent grafts were specifically selected for their capability to withstand overdilation up to a diameter of 12–16 mm. This feature is crucial for facilitating optimal adaptation to the broader proximal part and the narrower distal portion of the LSA. Reflecting the occurrence of endoleaks, it can be attributed to the insufficient insertion depth of the side branch into the LSA, resulting in inadequate sealing, which could be addressed with extension stent grafts.

An additional advantage of the FET–LSA prosthesis is the capability for antegrade insertion of guide wires via the brachial artery, allowing for potential future treatment of the thoracoabdominal aorta with branched or fenestrated endovascular prostheses.

In order to streamline FET implantation and facilitate the anastomosis to the LSA, Pichlmaier *et al.* [18] introduced a technique wherein the anastomosis to both the LSA and LCCA was executed using self-expanding covered Viabahn endograft (W.L. Gore & Associates, Flagstaff, AZ, USA). This technique, in combination with the distal anastomosis in zone 0, substantially reduced circulatory arrest time.

In addition to a refined surgical protocol, achieving optimal positioning of the FET prosthesis and employing effective methods for neural tissue protection is crucial for attaining successful outcomes. In our series, we utilized high moderate circulatory arrest (HMCA) with 27°C and bilateral SACP, combined with right axillary artery cannulation, as our approach to neural protection. The utilization of SACP alongside HMCA for complex aortic arch operations gained support from various authors [19–21]. A well-considered strategy for cerebral protection becomes paramount, particularly when facing a ‘circulatory arrest time’ of approximately 60 min. With the application of bilateral SACP, there exists no circulatory arrest time for the brain, as only a visceral circulatory arrest time occurs during the completion of the distal anastomosis, just prior to initiating distal aortic perfusion. While several publications have not identified significant differences among deep hypothermia, deep hypothermia combined with retrograde cerebral perfusion or antegrade cerebral perfusion with moderate hypothermia, it is worth noting that most of these studies were conducted in patients necessitating partial arch repair with circulatory arrest times of around 30 min [22–24]. Tsagakis *et al.* [25] could show that the combination of 4-site perfusion (bilateral carotid artery perfusion, LSA perfusion and distal aortic perfusion) with proximalization of the distal anastomosis in zone 2 significantly improved the outcome in FET surgery.

### Limitations of the study

With only 4 participants, it is challenging to draw statistically significant conclusions about the safety, efficacy, or potential side effects of the FET–LSA technique. The findings may not accurately represent the broader population due to limited diversity in factors such as age, gender, underlying pathology and medical history. Variability in individual responses may not be adequately captured with such a small sample size.

## CONCLUSION

In conclusion, we have successfully demonstrated the transition of an innovative FET prosthesis, previously tested in an experimental setting, to its inaugural clinical application in humans. The FET–LSA prosthesis holds promise in streamlining and expediting complex aortic arch surgery. However, further refinement is imperative to enhance the device’s usability and mitigate the incidence of LSA endoleaks. This study is only a preliminary step in the research process and must be followed by larger and more rigorous clinical trials to establish the treatment’s safety and effectiveness. Drawing from this initial experience, a modified FET prosthesis featuring an endovascular side branch for the LSA has been developed and will be evaluated in an FDA study in the United States and Europe.

## Supplementary Material

ezae302_Supplementary_Data

## Data Availability

The data underlying this article will be shared on reasonable request to the corresponding author.

## ABBREVIATIONS

CPB: Cardiopulmonary bypass
CT: Computed tomography
FET: Frozen elephant trunk
HMCA: High moderate circulatory arrest
LCCA: Left common carotid artery
LSA: Left subclavian artery
PAU: Penetrating atherosclerotic aortic ulcer
SACP: Selective antegrade cerebral perfusion
